# Enhancing Dementia Risk Prediction with Heart Rate and Machine Learning in the Canadian Longitudinal Study on Aging (CLSA)

**DOI:** 10.1002/alz70860_098393

**Published:** 2025-12-23

**Authors:** Shakiru A Alaka, So‐Fong C Ngan, Rebecca EK MacPherson, William Pickett, Christopher Chen, Newman Sze

**Affiliations:** ^1^ Brock University, St. Catharines, ON, Canada; ^2^ Brock University, St Catharines, ON, Canada; ^3^ Brock University, Centre for Neurosciences, St. Catharines, ON, Canada; ^4^ Memory, Ageing and Cognition Centre, National University Health System, Singapore, Singapore

## Abstract

**Background:**

Accurate and accessible risk assessment tools are essential for effective dementia management. The Cardiovascular Risk Factors, Aging, and Incidence of Dementia (CAIDE) model is the widely used tool to assess mid‐life dementia risk. This study aims to enhance the CAIDE model by incorporating resting heart rate (RHR), a simple and easily measurable cardiovascular factor, and applying machine learning methods to improve prediction accuracy.

**Methods:**

Data from 27,768 participants of comprehensive cohort in the Canadian Longitudinal Study on Aging (CLSA) were analyzed to predict 3‐year cognitive decline. Predictive models were developed using random forest and support vector machine algorithms. Performance was assessed using key metrics, including area under the receiver operating characteristic curve (AUC), sensitivity, specificity, Matthew's correlation coefficient (MCC), and Brier score. Internal cross‐validation was used to ensure model robustness.

**Results:**

Among the 18,013 participants with complete data for analysis, 516 (2.86%) exhibited cognitive impairment. Incorporating RHR into the CAIDE model led to a significant improvement in predictive accuracy. Random forest models with RHR achieved an AUC of 0.67 and an MCC of 0.32 in training data, compared to 0.65 and 0.29 in the test data. Similarly, support vector machines demonstrated a 2‐3% increase in both AUC and MCC with the inclusion of RHR.

**Conclusions:**

The addition of resting heart rate significantly improves the predictive accuracy of the CAIDE model, particularly when using machine learning methods. This highlights the potential of this approach to enhance traditional risk models, enabling more precise dementia risk assessments, closer monitoring, and the implementation of preventive strategies.

